# Supplementary figures and images for: Reversible domain closure modulates GlnBP ligand binding affinity
Source: PLoS One. 2022 Apr 21;17(4):e0263102. doi: 10.1371/journal.pone.0263102 (PMC9022810; doi:10.1371/journal.pone.0263102)

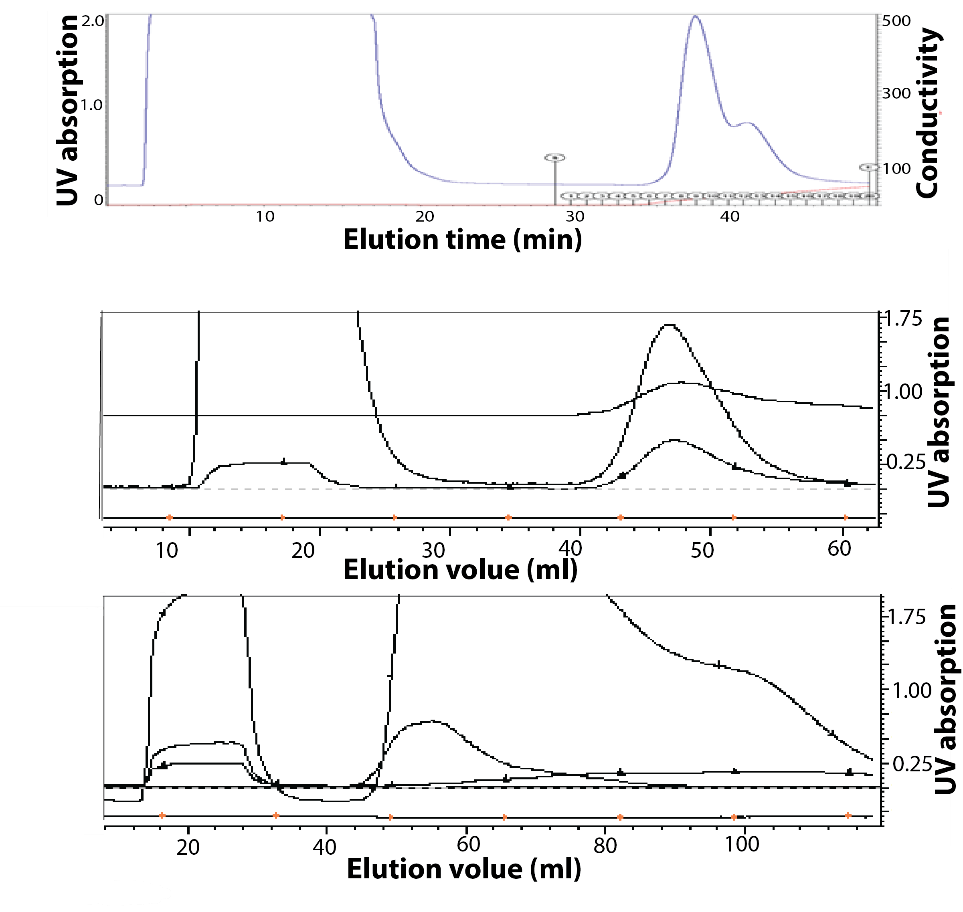

Supplement: S1 Fig — (TIF) [file pone.0263102.s001.tif]

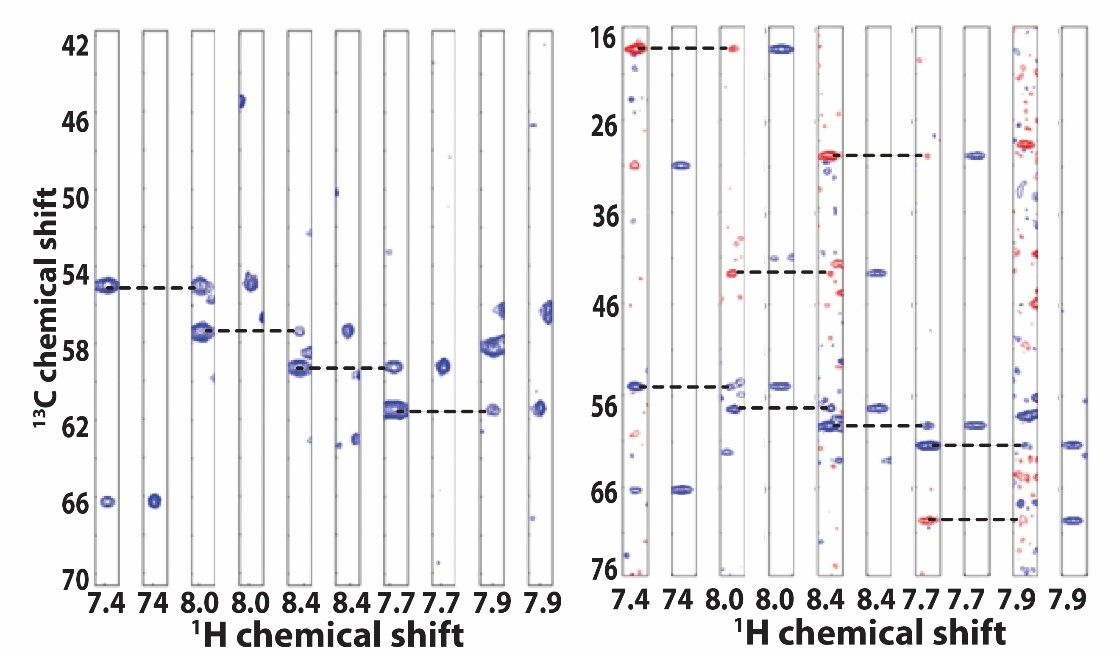

Supplement: S2 Fig — (TIF) [file pone.0263102.s002.tif]

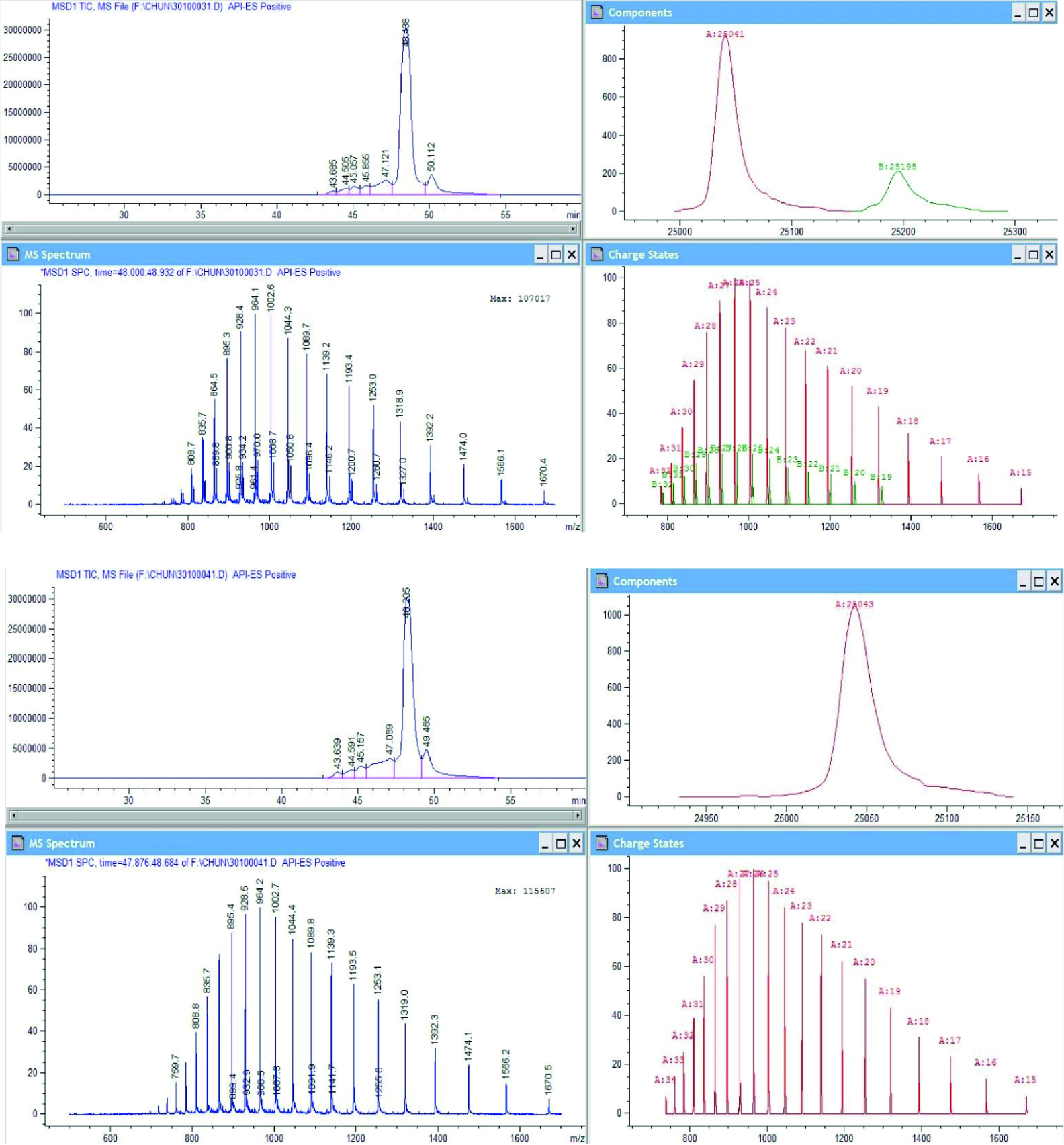

Supplement: S3 Fig — The corresponding molecular weight is 25041 and 25043. (TIF) [file pone.0263102.s003.tif]

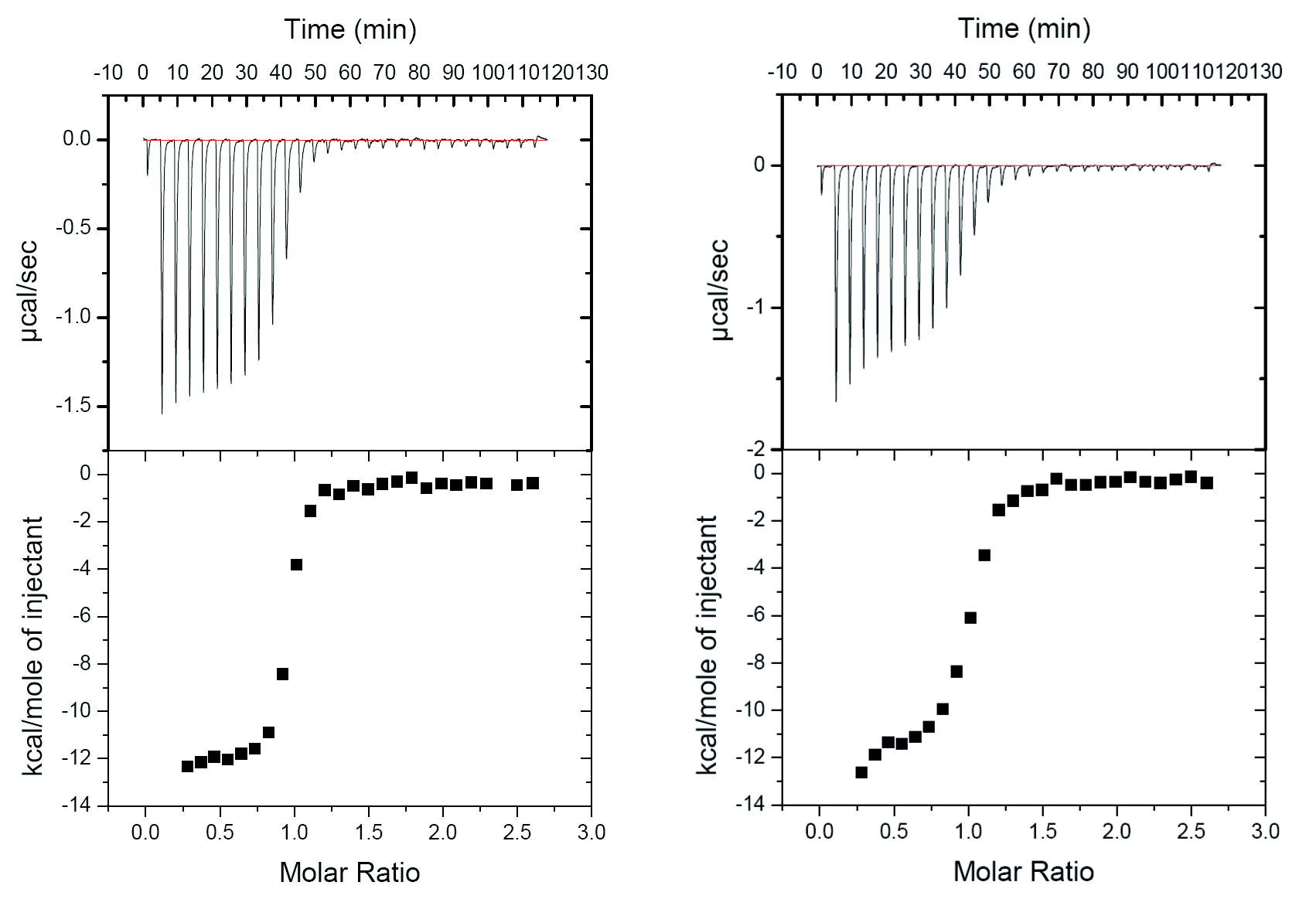

Supplement: S4 Fig — (TIF) [file pone.0263102.s004.tif]
