# Supplementary material for: Reversible domain closure modulates GlnBP ligand binding affinity
Source: PLoS One. 2022 Apr 21;17(4):e0263102. doi: 10.1371/journal.pone.0263102 (PMC9022810; doi:10.1371/journal.pone.0263102)
Supplement: Attachment — Submitted filename: Review_Combination of SAXS and NMR to monitor the conformation changes of E.pdf [file pone.0263102.s005.pdf]

Combination of SAXS and NMR to monitor the conformation changes of E. Coli glutamine binding protein upon ligand binding

The authors have investigated the configurational modifications of the glutamine binding protein (GlnBP) upon ligand binding by NMR and SAXS in order to determine the correlation between ligand binding affinity and domain closure. Dynamics of the hinge region, located between the two domains that constitute the protein, induce open-to-close conformation upon ligand binding and can be determined by variation of ligand concentration and mutants with linkers of different lengths. Although a complete structure determination of the conformational variations has not been achieved, the authors present a relevant structural characterization that contributes to the knowledge in the corresponding research field.

This article is of interest, well-written, and the experiments seem to be well-conducted, with few issues that need to be addressed that are listed below.

- The authors could use SAXS analysis to obtain envelope models for the different domain closure configurations and complement NMR data. Different methodologies for protein structure determination by NMR and SAXS conjugation can be implemented, as described at *Rodriguez-Zamora, P. "Conjugation of NMR and SAXS for flexible and multidomain protein structure determination: from sample preparation to model refinement." Progress in biophysics and molecular biology 150 (2020): 140-144.* If such data is available it should be included in the article, otherwise it would be recommended to mention the possibility of an NMR-SAXS conjugated analysis as a perspective for this work.

- A figure showing the protein hinge section and the linker introduction is required to illustrate the region of interest.

- Figure 5 is missing a color code that clarifies the results obtained by SAXS.

- Few grammar errors need to be corrected throughout the manuscript. A PDF copy with some of those errors highlighted is attached.
