# Supplementary material for: Reversible domain closure modulates GlnBP ligand binding affinity
Source: PLoS One. 2022 Apr 21;17(4):e0263102. doi: 10.1371/journal.pone.0263102 (PMC9022810; doi:10.1371/journal.pone.0263102)
Supplement: Attachment — Submitted filename: PONE-D-20-37100_reviewer.pdf [file pone.0263102.s006.pdf]

PLOS ONE

Combination of SAXS and NMR to monitor the conformation changes of E. Coli glutaminine binding protein upon ligand binding

--Manuscript Draft--

**Manuscript Number:**	PONE-D-20-37100
**Article Type:**	Research Article
**Full Title:**	Combination of SAXS and NMR to monitor the conformation changes of E. Coli glutaminine binding protein upon ligand binding
**Short Title:**	Combination of SAXS and NMR
**Corresponding Author:**	Dongmei Yu<br>Shandong University<br>Weihai, CHINA
**Keywords:**	small angle x-ray scattering (SAXS) ， NMR，Glutamine binding protein, isothermal titration calorimetry (ITC)
**Abstract:**	Glutamine binding protein (GlnBP) is an Escherichia Coli periplasmic binding protein, which binds and carries glutamine to the inner membrane ATP-binding cassette (ABC) transporter. GlnBP binds the ligand with affinity around 0.1μM measured by isothermal titration calorimetry (ITC) and ligand binding stabilizes protein structure shown by its increase in thermodynamic stability. Mutants with different linker length show different ligand binding affinity, which indicates that the propensity of domain closure determines the ligand binding affinity. Structural changes upon ligand binding are monitored by combination of small angle x-ray scattering (SAXS) and NMR spectroscopy. Detailed structure characterization of GlnBP contributes to a better understanding of ligand binding and provides the structural basis for biosensor design.
**Order of Authors:**	Dongmei Yu
	Qun Chen
	Jie Li
	Peiwu Qin
	Bo Qin
	Xiaobing Zuo
	Jin Chen
	Chengming Yang
**Additional Information:**	
**Question**	**Response**
**Financial Disclosure**<br><br>Enter a financial disclosure statement that describes the sources of funding for the work included in this submission. Review the submission guidelines for detailed requirements. View published research articles from *PLOS ONE* for specific examples.<br><br>This statement is required for submission and **will appear in the published article** if the submission is accepted. Please make sure it is accurate.	Prof. Peiwu Qin received National Natural Science Foundation of China (31970742, 81972854, 81802626, http://www.nsfc.gov.cn/english/site_1/index.html), and Science, Technology and Innovation Commission of Shenzhen Municipality (JSGG20191129110812, http://stic.sz.gov.cn/). The funders had no role in study design, data collection and analysis, decision to publish, or preparation of the manuscript.

Powered by Editorial Manager® and ProduXion Manager® from Aries Systems Corporation

**Unfunded studies**<br>Enter: *The author(s) received no specific funding for this work.*<br><br>**Funded studies**<br>Enter a statement with the following details:<br>• Initials of the authors who received each award<br>• Grant numbers awarded to each author<br>• The full name of each funder<br>• URL of each funder website<br>• Did the sponsors or funders play any role in the study design, data collection and analysis, decision to publish, or preparation of the manuscript?<br>• **NO** - Include this sentence at the end of your statement: *The funders had no role in study design, data collection and analysis, decision to publish, or preparation of the manuscript.*<br>• **YES** - Specify the role(s) played.<br><br>* typeset	
**Competing Interests**<br><br>Use the instructions below to enter a competing interest statement for this submission. On behalf of all authors, disclose any competing interests that could be perceived to bias this work—acknowledging all financial support and any other relevant financial or non-financial competing interests.<br><br>This statement **will appear in the published article** if the submission is accepted. Please make sure it is accurate. View published research articles from *PLOS ONE* for specific examples.	The authors have declared that no competing interests exist.

**NO authors have competing interests**<br><br>Enter: *The authors have declared that no competing interests exist.*<br><br>**Authors with competing interests**<br><br>Enter competing interest details beginning with this statement:<br><br>*I have read the journal's policy and the authors of this manuscript have the following competing interests: [insert competing interests here]*<br><br>* typeset	
**Ethics Statement**<br><br>Enter an ethics statement for this submission. This statement is required if the study involved:<br><br>• Human participants<br>• Human specimens or tissue<br>• Vertebrate animals or cephalopods<br>• Vertebrate embryos or tissues<br>• Field research<br><br>Write "N/A" if the submission does not require an ethics statement.<br><br>General guidance is provided below. Consult the submission guidelines for detailed instructions. **Make sure that all information entered here is included in the Methods section of the manuscript.**	N/A

**Format for specific study types**<br><br>**Human Subject Research (involving human participants and/or tissue)**<br>• Give the name of the institutional review board or ethics committee that approved the study<br>• Include the approval number and/or a statement indicating approval of this research<br>• Indicate the form of consent obtained (written/oral) or the reason that consent was not obtained (e.g. the data were analyzed anonymously)<br><br>**Animal Research (involving vertebrate animals, embryos or tissues)**<br>• Provide the name of the Institutional Animal Care and Use Committee (IACUC) or other relevant ethics board that reviewed the study protocol, and indicate whether they approved this research or granted a formal waiver of ethical approval<br>• Include an approval number if one was obtained<br>• If the study involved *non-human primates*, add *additional details* about animal welfare and steps taken to ameliorate suffering<br>• If anesthesia, euthanasia, or any kind of animal sacrifice is part of the study, include briefly which substances and/or methods were applied<br><br>**Field Research**<br><br>Include the following details if this study involves the collection of plant, animal, or other materials from a natural setting:<br>• Field permit number<br>• Name of the institution or relevant body that granted permission	
**Data Availability**<br><br>Authors are required to make all data underlying the findings described fully available, without restriction, and from the time of publication. PLOS allows rare exceptions to address legal and ethical concerns. See the PLOS Data Policy and FAQ for detailed information.	No - some restrictions will apply

A Data Availability Statement describing where the data can be found is required at submission. Your answers to this question constitute the Data Availability Statement and **will be published in the article**, if accepted.<br><br>**Important:** Stating 'data available on request from the author' is not sufficient. If your data are only available upon request, select 'No' for the first question and explain your exceptional situation in the text box.<br><br>Do the authors confirm that all data underlying the findings described in their manuscript are fully available without restriction?	
**Describe where the data may be found in full sentences. If you are copying our sample text, replace any instances of XXX with the appropriate details.**<br><br>• If the data are **held or will be held in a public repository**, include URLs, accession numbers or DOIs. If this information will only be available after acceptance, indicate this by ticking the box below. For example: *All XXX files are available from the XXX database (accession number(s) XXX, XXX.*).<br>• If the data are all contained **within the manuscript and/or Supporting Information files**, enter the following: *All relevant data are within the manuscript and its Supporting Information files.*<br>• If neither of these applies but you are able to provide **details of access elsewhere**, with or without limitations, please do so. For example:<br><br>*Data cannot be shared publicly because of [XXX]. Data are available from the XXX Institutional Data Access / Ethics Committee (contact via XXX) for researchers who meet the criteria for access to confidential data.*<br><br>*The data underlying the results presented in the study are available from (include the name of the third party*	BMRB ID: 50310

and contact information or URL).<br>• This text is appropriate if the data are owned by a third party and authors do not have permission to share the data.<br><br>* typeset	
Additional data availability information:	

Powered by Editorial Manager® and ProduXion Manager® from Aries Systems Corporation

Manuscript Click here to access/download;Manuscript;manuscript202011.pdf

Combination of SAXS and NMR to monitor the conformation changes of *E. Coli* glutaminine binding protein upon ligand binding

Qun Chen[1,2,†], Jie Li[3,†], Xiaobing Zuo[4], Jin Chen[5], Peiwu Qin [2,*], Bo Qin[6,*], Chengming Yang[7*],Dongmei Yu [1,*]

[1] School of Mechanical, Electrical & Information Engineering, Shandong University, Weihai, Shandong Province, 264209, China

[2] Center of Precision Medicine and Healthcare, Tsinghua-Berkeley Shenzhen Institute, Shenzhen, Guangdong Province, 518055, China

[3] Kunming Dog Base of Police Security, Ministry of Public Security, Kunming, Yunnan Province, 650204, China

[4] X - ray Science Division, Argonne National Laboratory, Argonne, IL, 60439, USA

[5] Institute of chinese medical sciences, University of Macau, Macau, 999078, China

[6] Shenzhen Aier Eye Hospital, Futian District, Guangdong Province, 518033, China

[7] University of Science and Technology Hospital, Shenzhen, Guangdong Province, 518000, China

*Authors to whom correspondence should be addressed. Correspondence: yudongmei@sdu.edu.cn (Dongmei Yu).

ABSTRACT: Glutamine binding protein (GlnBP) is an *Escherichia Coli* periplasmic binding protein, which binds and carries glutamine to the inner membrane ATP-binding cassette (ABC) transporter. GlnBP binds the ligand with affinity around 0.1μM measured by isothermal titration calorimetry (ITC) and ligand binding stabilizes protein structure shown by its increase in thermodynamic stability. Mutants with different linker length show different ligand binding affinity, which indicates that the propensity of domain closure determines the ligand binding affinity. Structural changes upon ligand binding are monitored by combination of small angle x-ray scattering (SAXS) and NMR spectroscopy. Detailed structure characterization of GlnBP contributes to a better understanding of ligand binding and provides the structural basis for biosensor design.

INTRODUCTION

In Gram-negative bacteria, periplasmic ATP-binding cassette (ABC) transporter systems are responsible for transporting a broad variety of nutrients across the cytoplasmic membrane. ABC transporter consists of three components: a periplasmic binding protein, which shuttles the ligands through the outer membranes into the periplasmic spaces; an integral membrane protein complex that provides the transmembrane pathway; and two cytoplasmic nucleotide-binding domains that provide the driving power by hydrolyzing ATP molecules. The interaction between the protein transporter and ligand activates the energy-coupling transportation pathway and results in the opening of a channel and the subsequent translocation of the ligand across the cytoplasmic membrane (*1, 2*).

The first periplasmic binding protein was discovered by Pardee in 1996 (*5*). Since then, many periplasmic binding proteins have been characterized and their structures have been studied extensively by x-ray crystallography and solution NMR (*3, 4, 6, 7*). The periplasmic binding proteins of ABC transporters from Gram-negative bacteria possess a common architecture (Fig. 1). Glutamine binding protein (GlnBP), ATP-binding cassette (ABC) transporter, in this study is *Escherichia Coli* periplasmic binding protein of L-glutamine, which binds and carries glutamine to the inner membrane. The crystal structures of ligand-free and bound GlnBP show obvious conformational change upon ligand binding by structural alignment (*3, 4*). GlnBP is classified in the same Structural Classification of Proteins SCOP family (phosphate- binding protein-like) as human GluR2, but shares 17% sequence identity with GluR2. GlnBP is comprised of two domains linked by a flexible “hinge” region, whose movement induces open-to-close conformation upon ligand binding. GlnBP undergoes conformational change due to the hinge bending and twisting motion (*8*). The deep central cleft between domains provides the ligand-binding site with sub-micromolar affinity. The conformational changes upon ligand binding have been studied by the combination of chemical shift perturbation and ^{15}N-^{1}H scalar coupling (*9*). The aliphatic side chain of the glutamine is sandwiched in a hydrophobic pocket formed between Phe13 and Phe50, which has 21 van der Waals contacts with GlnBP Lys115 and His156. These interactions are unique to GlnBP among known amino acid binding proteins, which apparently contribute to the ligand binding specificity of GlnBP. The hydrogen-bond networks increase the stability of GlnBP-ligand complex (*4*).

Ligand-bound GlnBP triggers the conformational change of downstream membrane transporter, but not ligand free GlnBP. Ligand binding modifies GlnBP’s binding property with inner membrane transporter. GlnBP structural information is required to delineate GlnBP binding specificity and dynamic property. The use of GlnBP as glutamine biosensor has been proposed. However, the detailed knowledge of protein dynamics, binding affinity, and thermal stability is required for the development of sensor (*8*). Here, we present the first small angle x-ray scattering study on GlnBP to monitor the gradual ensemble structural changes upon ligand binding. Ligand is removed completely from GlnBP by GdnHCl and EGTA; then GlnBP is refolded on desalting column to remove GdnHCl. Ligand binding affinity is measured by isothermal titration calorimetry. Mutants are created with site-directed mutagenesis to change the ligand affinity by introducing a linker on the opposite site of ligand binding pocket. The molecular weights of mutants are verified by mass spectrometry. Thermal stability is studied with urea CD titration and Gibbs free energy is extracted from the CD urea titration curve. GlnBP conformational changes have been studied by NMR (*6, 9*), but the complete backbone assignment is still missing. Sedimentation velocity experiments of analytical ultracentrifuge conclude that GlnBP is monomeric and interference data shows the presence of aggregates

ranging from ~0.1% at 83 μM to ~0.2% at 1480 μM. The data presented in this article is a further step towards understanding of the ligand binding, structural changes, and thermodynamics of GlnBP.

EXPERIMENTAL METHODS

GlnBP Protein Expression, Purification, and Removal of Ligand. GlnBP gene is cloned into pET11a vector and the sequence is verified by DNA sequencing. Plasmid with GlnBP gene is transformed into BL21 star (DE3) competent cells for protein expression. Bacteria are cultured overnight in a 5 ml LB medium containing 100 μg/ml of ampicilin at 37 °C, and then cell pellet（minimize the volume of LB medium） is transferred into 1 L M9 minimal medium (6.78 g Na_2HPO_4, 3.0 g KH_2PO_4, 1 g NaCl, 2 g Glucose, 1 g ^{15}N $NHCl_4$, 2 mM $MgSO_4$, 0.2 mM $CaCl_2$, 1x MEM vitamin mix, 1x trace element solution, 100 μg ampicilin per liter medium) at 37 °C until OD_{600} reaches around 0.8. 1 mM IPTG is used to induce protein express for 3 hours at 37°C. Cells are harvested at 5000 g for 30 min at 4 °C. The cell pellets are resuspended in the buffer (10 mM sodium acetate, pH 5.5) and broken by microfluidizer at 1200 psi. Clear supernatant is collected by centrifuge at 12,000 rpm for 30 min. Protein is purified by cation exchange (CM column) and GlnBP collections are pooled and buffer exchanged for gel filtration with 10 mM Tris and 300 mM NaCl. The fractions with GlnBP from gel filtration are pooled and exchanged to 10 mM Tris buffer with Amicon Ultra-10K (10,000 Da MWCO) device. Concentrated GlnBP run through cation exchange chromatography and elute with a linear gradient of 0-500 mM NaCl. The identity and integrity of the final protein is confirmed by SDS-PAGE. The ligand glutamine is dissociated from protein by treatment with 6 M guanidine hydrochloride at room temperature for 2 hours. Desalting column is equilibrated with 2 M and 6 M GdnHCl and GlnBP is run thought the column to remove glutamine. The desalting column is thoroughly washed and equilibrated with 10 mM Tris. Guanidine is removed by running the desalting column with 10 mM Tris. 10 mM Tris (pH 6.8) buffer will be used for further NMR and ITC experiments, but 10 mM phosphate buffer will be used for CD urea titration because of the strong absorbance of Tris buffer.

Isothermal Titration Calorimetry. GlnBP is dialysed into buffer A (10 mM Tris at pH 7.2). Glutamine is dissolved into buffer A. Background titration is performed to check the dilution effect of glutamine. For optimal measurement of ITC, the product of protein concentration and association constant should stay in the range between 1 to 100. The protein concentration is 10-30 uM and ligand is 10 times concentrated about 300 uM. Around 1.8 ml protein is loaded into the sample cell with long syringe and 292 ul ligand is loaded into injection needle with stirring to remove bubbles. First step injects 2 ul ligand into protein solution, and then 10 ul ligand is injected each time with 4 min delay between injections. The plot of DP (different power) with respect to the time is drawn by MicroCal software and thermal parameter and binding affinity are extracted by fitting the data.

Circular Dichroism. Urea denaturation is monitored by circular dichroism (CD) with an AVIV Model 62DS spectrometer. Experiments are conducted in 10 mm cuvetts with constant 2.0 mL volume. About 5-6 μM protein in 10 mM phosphate buffer (pH 7.5) is used in all the assays. Wavelength from 190 nm to 230 nm is scanned to find appropriate wavelength to monitor the protein structural changes. The dynode is kept below 500. The urea titration is from 0 M to 8 M with 0.1 M increment for 80 shots and 30 s averaging time. The sample is stirred at 78 rpm during the process of titration. The data is analyzed by Origin. The ellipticity data, corrected for dilution, are fit to equation (1):

$$y = \frac{y_n + m_n[\text{urea}] + (y_u + m_u[\text{urea}]) \times \exp\left(-\left(\frac{\Delta G_0 - m[\text{urea}]}{RT}\right)\right)}{1 + \exp\left(-\left(\frac{\Delta G_0 - m[\text{urea}]}{RT}\right)\right)} \quad (1)$$

where m_n and y_n are the slope and intercept, respective, for the pre-transition baseline; y_u and m_u are the slope and intercept for the post-transitional baseline; ΔG_0 is the Gibbs energy change for unfolding in the absence of urea; m describes the sensitivity of conformational free energy to urea concentration (*10*).

Small Angle X-ray Scattering. SAXS is performed at Argonne National Laboratory sector 12-ID-B. Data are collected at room temperature on a 1024 x 1024 pixel CCD detector with sample-to-detector distance of 1 m and the transmission intensity is measured with a PIN diode beamstop. Protein solution scattering is conducted with concentration of 6.5 mg/ml in 10 mM Tris, pH 7.5. Background scattering from buffer is subtracted from all samples. The zero angle scattering intensity I(0) and overall radius of gyration R_g are obtained from a Guinier approximation to the low-q region of the scattering profiles satisfying the condition, Q_{max}* R_g < 1.3. The P(r) functions have the characteristic bell shape of globular complexes with well-defined maximum diameter size for all the samples prepared. *I*(*q*) is the scattered X-ray intensity per unit solid angle and *q* is the amplitude of the scattering vector, given by $4\pi(\sin\theta)/\lambda$, where 2θ is the scattering angle and λ is the wavelength of the scattered X-rays (0.98 Å). ΔP(r) manifests statistical significant variation in the distribution of the frequency of vectors lengths in the P(r) functions (*11*).

NMR spectroscopy

NMR experiments are performed at 310 K on a Bruker Ultrashield 800 MHz and 500 MHz with a z-gradient cryoprobe. Protein samples are concentrated to 500 μM with 10 mM Tris (pH 7.5) and 7% D_2O is added. All NMR spectra are processed with software package NMRPipe (*12*) and analyzed using NMRView (*13*). Quadrature detection in the indirect dimensions is achieved either with States-TPPI or Echo/antiecho methods. Sequential backbone assignments are obtained from the following two- and three-dimensional experiments: 2D ^{1}H-^{15}N HSQC, 3D HNCO, HNCA, HN(CO)CACB, and HNCACB (*14*).

RESULTS

The structural and thermodynamics change of GlnBP upon ligand binding are investigated by ITC, CD, NMR, and SAXS. Protein is purified by ion exchange and gel filtration chromatography. The purity is check by SDS-PAGE, which shows a single band at 25 KDa. Overexpressed GlnBP is a mixture of ligand-free and bound GlnBP, which could be inferred from the shoulder peak on ion exchange chromatography. The collections are pooled and treated with guanidine hydrochloride to disrupt the ligand binding pocket and release ligand. The sample is loaded into the desalting column while maintaining 2 M guanidine during the process of separation. Then, we exchange the HPLC buffer with 10 mM Tris and run the sample through desalting column again to remove guanidine.

The ligand binding affinity is measured with ITC for wild-type and mutants of GlnBP. Mutants with different ligand binding affinity are created by introducing a polypeptide linker composed of different numbers of alanine residue from 0 to 5 residues. The smaller side chain of alanine reduces the possibility of creating steric hindrance. The length of the linker between N170C (Asn 170 was mutated to Cys) and C terminal cysteine controls the magnitude of domain closure based on our structural analysis. The formation of disulfide bond is verified by Ellman's assay and mass spectrometer. The formation of disulfide bond decreases molecular weight by 2 due to the formation of new polypeptide bond, which can be verified by the molecular weight of GlnBP mutant with four alanine liner (Fig. 3a,b,d). Reduction of disulfide bond changes the molecular weight from 25043 to 25041 (Fig. 3c, e). The correlation between domain closure and ligand binding is observed since shorter linker mutants have a lower binding affinity, which indicates that ligand binding triggers the protein conformational change and induces domain closure to create a perfect binding site for ligand.

NMR methods have been developed to measure structural changes of GlnBP (*9*). However, the complete backbone assignment of ligand-free GlnBP is still missing and 92% backbone assignment is achieved in this study. Assignment starts from residues with unique chemical shifts like alanine (average 19.11 ppm for Cβ), serine and threonine (average 63.74 ppm and 69.62 ppm for Cβ respectively), and glycine (average 45.38 ppm for Cα). Connectivity between residues was established initially using MARS (*15*) in an iterative manner, adding or adjusting assignments manually. Structural changes of GlnBP upon ligand binding are shown by dramatic change in heteronuclear single quantum correlation spectroscopy (HSQC) spectra of ligand-free and bound GlnBP (BMRB ID: 50310). Residues that are far from ligand binding sites also show chemical shift perturbation, which means that the binding event not only change the structure of binding pocket but change the whole protein structure（Fig. 2a, b）.

Sedimentation velocity of analytical ultracentrifuge is used to check the aggregates of GlnBP at different concentrations. GlnBP is primarily monomeric, but inference data shows the presence of aggregates and the amount

of aggregate appears to depend on the loading concentration from 0.1% at 83 μM to 2% at 1480 μM. The protein concentration for SAXS is 197 μM in 10 mM Tris, which corresponds to 4.9 mg/ml. Guinier analysis is used to determine the overall radius of gyration, Rg, and the concentration-normalized forward scattering intensity, I(0)/c, which are functions of spatial size and molecular mass respectively. The Guinier plots are linear at the low q-range at all concentrations, indicating that GlnBP has a well-defined size under aqueous condition and radiation-induced aggregation is not an issue. Rg and I(0)/c, determined from linear fitting to the data over the q range satisfying the q * Rg < 1.3 condition (q = 0.0146 to 0.0416 $Å^{-1}$), display very little concentration dependence, indicating that no change in oligomeric state occurs over the investigated concentration range (Fig. 5a, b). Structural changes of GlnBP are also shown by SAXS ΔP(r) curves. With the increase in ligand concentration, the gradual changes are shown on ΔP(r) curves (Fig. 5c, d).

Thermo stability is measured with urea CD titration (Fig. 4a, b). The difference of Gibbs free energy between ligand-free and bound GlnBP is 59.82 kJ/mol and the energy of hydrogen bond is 5-30 kJ/mol, which indicates that ligand binding introduces extra hydrogen bonds and van der Walls interactions that account for the energy difference. Tertiary structure changes dramatically but secondary structures are maintained, which explain the higher energy level of ligand-free GlnBP. GlnBP is rigid with $\{^1H\}^{15}N$ NOE values around 0.9 except the N and C terminals and some residues that show slightly higher flexibility (Fig. 4).

DISCUSSION

GlnBP is a soluble glutamine binding protein swimming in the periplasmic space and escorts free glutamine into membrane transporter. Ligand binding to GlnBP triggers a large-scale conformational change and two domains close to pack the glutamine into the ligand binding pocket. The ligand binding affinity determines the efficiency of transportation *in vivo* and the application of GlnBP in the field of biosensors. Dynamics of GlnBP have been characterized by NMR (*6*) and molecular dynamics simulation (*16*). However, the correlation of structure and ligand binding affinity remains elusive. Mutants of GlnBP with different binding affinity have been created and show the correlation between ligand binding affinity and domain closure, which is the major conformational change upon ligand binding.

The structure of GlnBP is strikingly similar to the ligand binding core (S1S2) of ionotropic glutamate receptor. Ionotropic glutamate receptors (iGluR) are major excitatory receptors in the vertebrate central nervous system. The ligand-binding core of iGluR is formed by polypeptide fragments S1 and S2, which could be coupled with a 13-residue liner to form a new construct S1S2. S1S2 has the same binding property as wild-type protein. The homotetrameric structure of intact iGluR was resolved recently and it demonstrates that S1S2 construct has

identical structure as the ligand-binding core of intact receptor. Structural and dynamic information obtained from GlnBP could shed light on the function of iGluR and provides hints about the structural conservation even if their sequences have diverged dramatically.

Acknowledge

This work was funded by National Natural Science Foundation of China (31970742, 81972854, 81802626), Science, Technology and Innovation Commission of Shenzhen Municipality (JSGG20191129110812), and Southern University of Science and Technology Hospital Dean Research Fund (2020-A4).

Legends for Figs.

Fig. 1: Model of glutamine transportation and structure of GlnBP. (a) Ligand-bound GlnBP interacts with membrane ABC transporter and induces the transportation of glutamine into the cytoplasm. (b) GlnBP has two domains and the interdomain rotation is demonstrated by the transition from green to red structure model while maintaining the position of another domain when glutamine binds to GlnBP.

Fig. 2: (a) Overlay of HSQC spectra for ligand-bound and free GlnBP. The red spectrum represents ligand-bound GlnBP and the blue one is GlnBP in ligand-free form; (b) HSQC of ligand-free GlnBP with the assignments.

Fig. 3: ITC of ligand binding. (a) ITC for wild-type GlnBP; (b) ITC for GlnBP 2A mutant and (c) with the addition of reducing reagents (DTT); (d) ITC for GlnBP 3A mutant and (e) with the addition of reducing reagents (DTT).

Fig. 4: CD Urea titration. (a) 10 M urea was titrated into 5 M ligand bound and (b) ligand free GlnBP solution.

Fig. 5: Small angle X-ray scattering of GlnBP. (a) Offset of I(q) vs q plot for GlnBP with different concentrations of ligand; (b) Overlay of I(q) vs q plots for GlnBP with different concentrations of ligand; (c) Pair distance distribution function P(r) plot of GlnBP with different concentrations of ligands; (d) ΔP(r) shows the difference of conformational changes when different concentrations of ligand were titrated into the GlnBP protein solution.

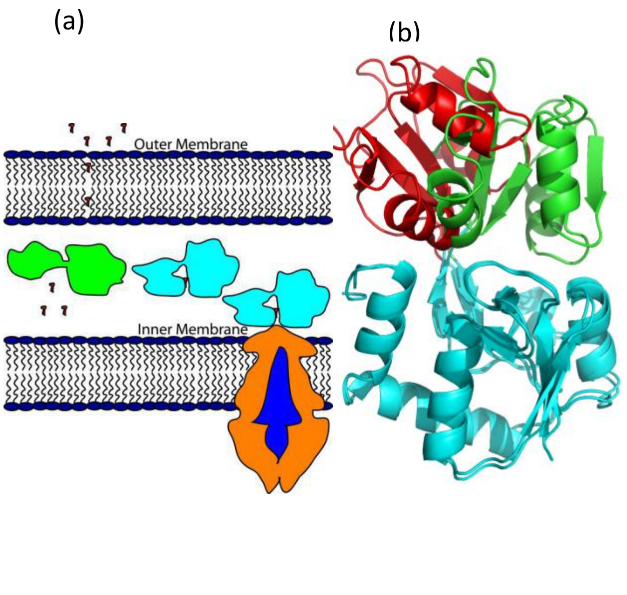

Fig. 1

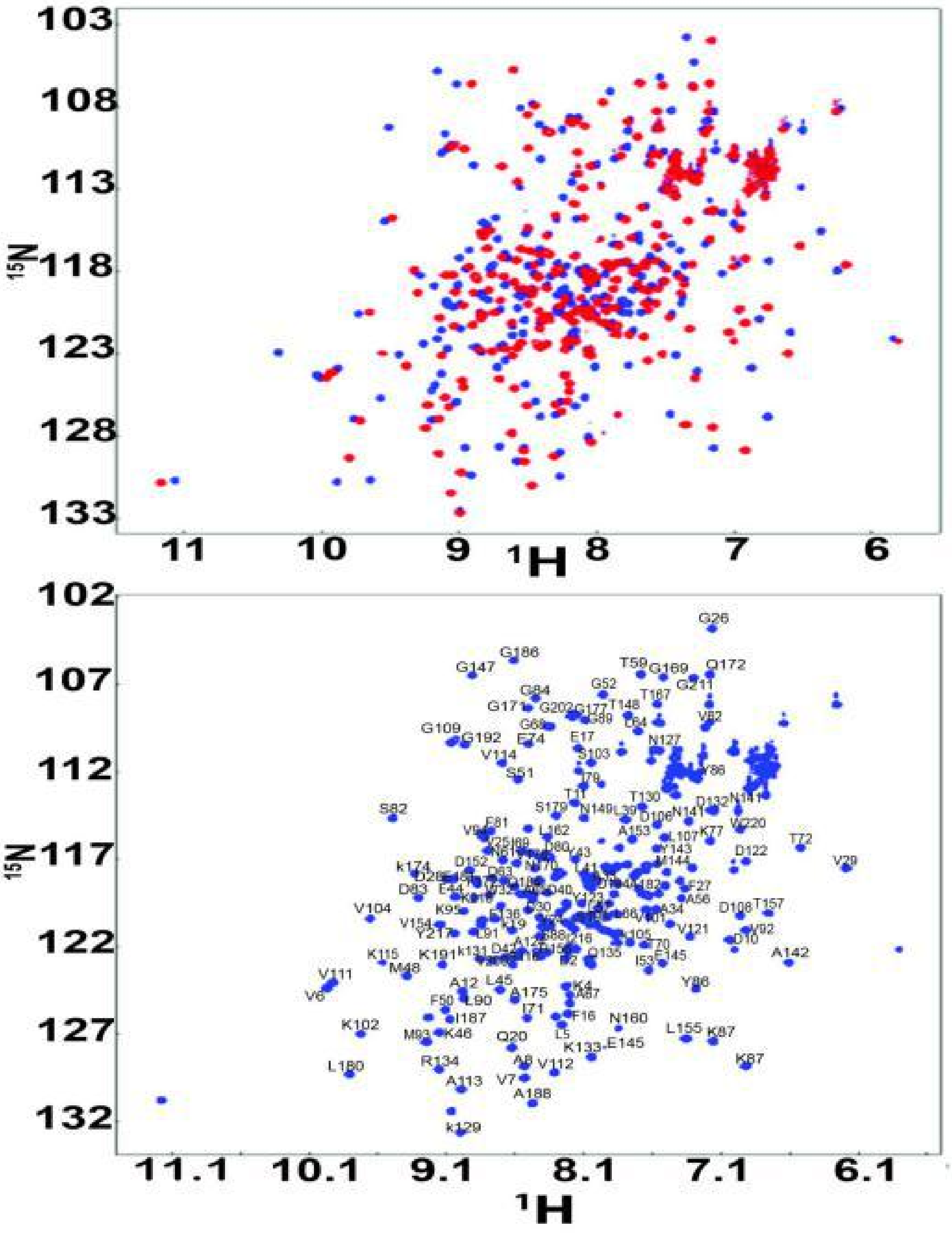
103
108
113
118
123
128
133
15N
11
10
9
1H
8
7
6
102
107
112
117
122
127
132
15N
11.1
10.1
9.1
8.1
7.1
6.1
1H
G26
G186
G147
T59
G169
Q172
G52
G211
G84
T187
G171
T148
G109
G192
E74
E17
N127
V114
S103
S51
Y86
T130
N141
S82
D122
T72
V20
V104
K95
V154
T157
D10
V92
V121
A142
K115
K191
M48
Q135
V111
V6
A12
L45
A175
A87
F50
L90
F16
I187
I71
N160
K102
M93
K46
Q20
L5
L155
K87
K133
E145
R134
V112
L180
A113
V7
A188
k129

Fig. 2

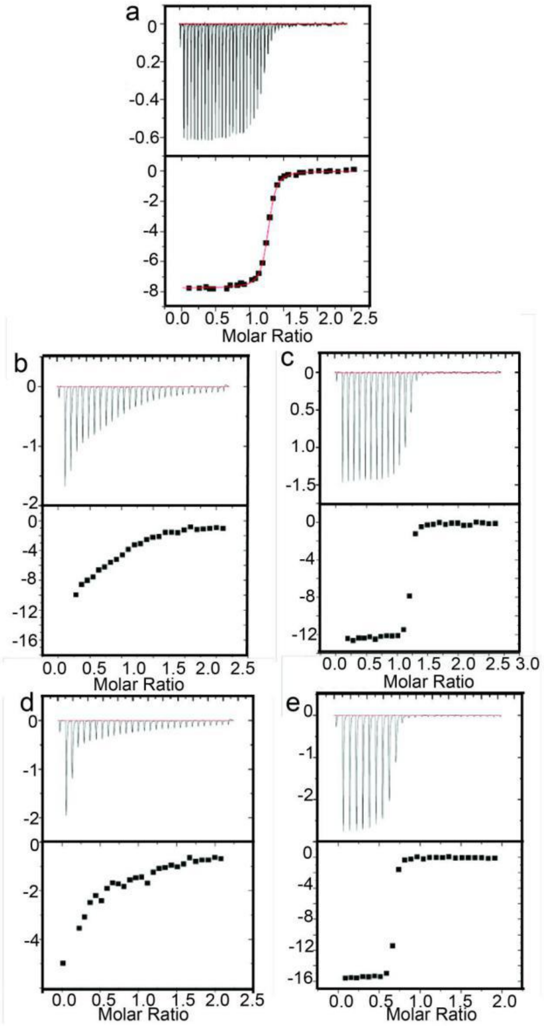
a
b
c
d
e
Molar Ratio
Molar Ratio
Molar Ratio
Molar Ratio
Molar Ratio

Fig. 3

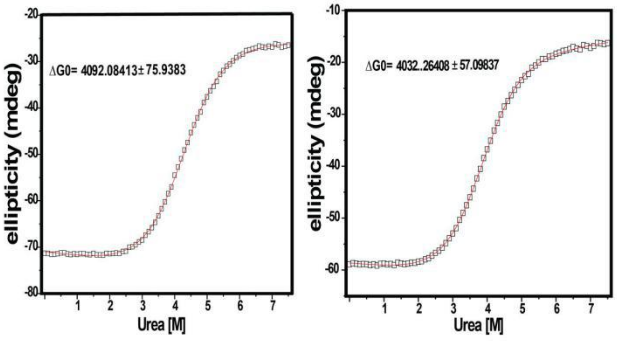
ΔG0= 4092.08413±75.9383
ellipticity (mdeg)
Urea [M]
ΔG0= 4032..26408 ± 57.09837
ellipticity (mdeg)
Urea [M]

Fig. 4

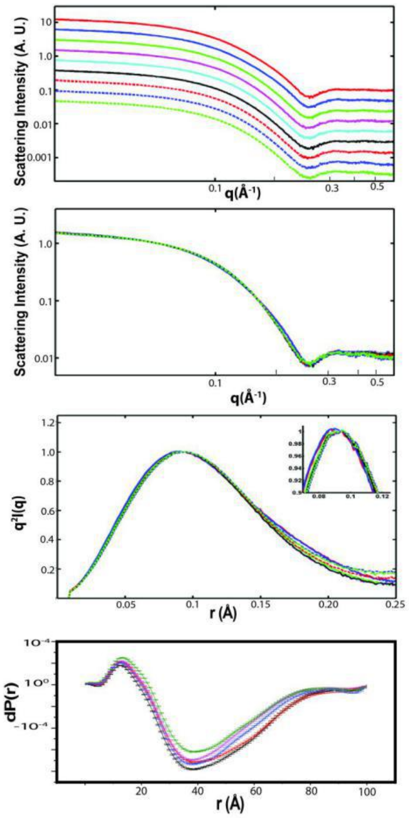

Fig. 5

References

1. Grunau, A., Geraki, K., Grossmann, J. G., and Gutierrez, A. (2007) Conformational dynamics and the energetics of protein--ligand interactions: role of interdomain loop in human cytochrome P450 reductase, *Biochemistry 46*, 8244-8255.
2. Hu, Y., Fan, C. P., Fu, G., Zhu, D., Jin, Q., and Wang, D. C. (2008) Crystal structure of a glutamate/aspartate binding protein complexed with a glutamate molecule: structural basis of ligand specificity at atomic resolution, *J Mol Biol 382*, 99-111.
3. Hsiao, C. D., Sun, Y. J., Rose, J., and Wang, B. C. (1996) The crystal structure of glutamine-binding protein from Escherichia coli, *J Mol Biol 262*, 225-242.
4. Sun, Y. J., Rose, J., Wang, B. C., and Hsiao, C. D. (1998) The structure of glutamine-binding protein complexed with glutamine at 1.94 A resolution: comparisons with other amino acid binding proteins, *J Mol Biol 278*, 219-229.
5. Linton, K. J. (2007) Structure and function of ABC transporters, *Physiology (Bethesda) 22*, 122-130.
6. Bermejo, G. A., Strub, M. P., Ho, C., and Tjandra, N. (2010) Ligand-free open-closed transitions of periplasmic binding proteins: the case of glutamine-binding protein, *Biochemistry 49*, 1893-1902.
7. Yu, J., Simplaceanu, V., Tjandra, N. L., Cottam, P. F., Lukin, J. A., and Ho, C. (1997) 1H, 13C, and 15N NMR backbone assignments and chemical-shift-derived secondary structure of glutamine-binding protein of Escherichia coli, *J Biomol NMR 9*, 167-180.

8. Pang, A., Arinaminpathy, Y., Sansom, M. S., and Biggin, P. C. (2003) Interdomain dynamics and ligand binding: molecular dynamics simulations of glutamine binding protein, *FEBS Lett 550*, 168-174.
9. Ma, J., Gruschus, J. M., and Tjandra, N. (2009) 15N-1H scalar coupling perturbation: an additional probe for measuring structural changes due to ligand binding, *J Am Chem Soc 131*, 9884-9885.
10. Henzl, M. T., and Ndubuka, K. (2007) Low-affinity signature of the rat beta-parvalbumin CD site. Evidence for remote determinants, *Biochemistry 46*, 23-35.
11. Fenton, A. W., Williams, R., and Trewhella, J. (2010) Changes in small-angle X-ray scattering parameters observed upon binding of ligand to rabbit muscle pyruvate kinase are not correlated with allosteric transitions, *Biochemistry 49*, 7202-7209.
12. Delaglio, F., Grzesiek, S., Vuister, G. W., Zhu, G., Pfeifer, J., and Bax, A. (1995) NMRPipe: a multidimensional spectral processing system based on UNIX pipes, *J Biomol NMR 6*, 277-293.
13. Johnson, B. A. (2004) Using NMRView to visualize and analyze the NMR spectra of macromolecules, *Methods Mol Biol 278*, 313-352.
14. Ferentz, A. E., and Wagner, G. (2000) NMR spectroscopy: a multifaceted approach to macromolecular structure, *Q Rev Biophys 33*, 29-65.
15. Jung, Y. S., and Zweckstetter, M. (2004) Mars -- robust automatic backbone assignment of proteins, *J Biomol NMR 30*, 11-23.
16. Loeffler, H. H., and Kitao, A. (2009) Collective dynamics of periplasmic glutamine binding protein upon domain closure, *Biophys J 97*, 2541-2549.
