# Supplementary material for: Reversible domain closure modulates GlnBP ligand binding affinity
Source: PLoS One. 2022 Apr 21;17(4):e0263102. doi: 10.1371/journal.pone.0263102 (PMC9022810; doi:10.1371/journal.pone.0263102)
Supplement: Attachment — Submitted filename: review.docx [file pone.0263102.s007.docx]

The manuscript by Chen et al. described the structure changes of addition mutant of E. coli. Glutamine binding protein (GlnBP) upon ligand (glutamine) binding. The experimental methods of ITC, NMR, SAXS etc. were applied. The conclusion was the change of binding affinity was associated with quaternary structure change (inter-domain) caused by the lengthened linker mutant. While the inter-domain reorganization may contribute to the binding affinity changes, the contribution is one of many factors that could affect binding affinity. Potentially, electrostatic and hydrophobic interactions between protein side chains and Gln should dominate the interaction, not discussed in the manuscript. The authors don’t seem to have a clear experimental design or conclusion. In general, for a study like this, the hypotheses should be raised at the beginning, followed by experimental demonstration, result interpretation and final conclusion. Now, the manuscript lacks coherent reasoning or conclusion. In the end, there is no take home message from the study. The manuscript is highly immature and should not be published in the current format. Major issues are listed below.

1. The result did not provide any quantitative correlation between ITC results and any others like SAXS or NMR. Therefore, the science advance on this binding topic is minimal.
2. The scattering of “Results” part was not acceptable, where sections were missing. The authors appeared to be random in narratively describing the results. Normally, sections of mutation selection, structure changes, correlation between xxx and yyy etc, should be in “Results”. For a biophysics study like this, a table of GlnBP protein of WT, mutant 1, mutant 2 … should be presented. This should be followed by structural characterization (NMR., SAXS, SV-AUC) at free and binding states of each protein. The authors should then infer from analytical results on the structural change of GlnBP at secondary, tertiary, quaternary structure levels. For example, NMR chemical shift could be sensitive to secondary and tertiary structure, relaxation could be sensitive to quaternary and oligomerization, SAXS could be sensitive to quaternary or domain structure, SV-AUC is for oligomerization. All of these standard reasonings are missing in the manuscript.
3. The 1st paragraph in “Results” belongs to method description.
4. Page 2 Paragraph 1, the statement “two domain linked by a flexible “hinge” region” was not correct. The 2 domains were linked by 2 rigid beta-strands per crystal structure.
5. The method section missed the description of SV-AUC method.
